# Infant Feeding Practices and Metal Concentrations in Children’s Blood

**DOI:** 10.1001/jamanetworkopen.2023.48230

**Published:** 2023-12-18

**Authors:** Anna R. Smith, Ruwan Thilakaratne, Pi-I D. Lin, Sheryl L. Rifas-Shiman, Marie-France Hivert, Emily Oken, Andres Cardenas

**Affiliations:** 1 Department of Epidemiology and Population Health, Stanford Medicine, Stanford, California; 2 Division of Epidemiology, University of California Berkeley School of Public Health, Berkeley; 3 Division of Chronic Disease Research Across the Lifecourse, Department of Population Medicine, Harvard Medical School and Harvard Pilgrim Health Care Institute, Boston, Massachusetts

## Abstract

This cohort study assesses the association between 4 infant feeding practices and concentrations of 8 nonessential and 7 essential metals in red blood cells.

## Introduction

Human milk provides complete nutrition and immune support for infants; infant formula is an alternative. Nonessential metals (eg, cadmium, lead, and mercury) partition into human milk fractions via exogenous and endogenous sources^1^ and may contaminate formula.^2^ Essential metals, including magnesium, manganese, selenium, and zinc, enter human milk from maternal diet or environmental pollution and are added to formula. Human milk and formula are known sources of metals (eg, lead) in children’s blood.^3^ We evaluated the association between infant feeding practices at 6 months and early childhood blood metal concentrations.

## Methods

We recruited pregnant participants of Project Viva, a prospective cohort study, during their first prenatal visit at Atrius Harvard Vanguard Medical Associates from 1999 to 2002. We assessed maternal-reported infant feeding practices at 6 months, categorizing them as exclusive breastfeeding, mixed feeding (breast milk and formula), fully weaned from breast milk to formula, or never breastfed. We quantified early childhood concentrations (nanograms of metal per gram of red blood cells [RBCs]) of 8 nonessential metals (arsenic, barium, cadmium, cesium, lead, mercury, strontium, tin) and 7 essential metals (cobalt, copper, magnesium, manganese, molybdenum, selenium, zinc) in children’s blood using triple quadrupole inductively coupled plasma mass spectrometry. We used linear regression to ascertain the percentage difference in metal concentration associated with milk source at 6 months, controlling for maternal age at enrollment, self-reported race and ethnicity, education level, annual household income, and parity as well as child assigned sex at birth and age at blood draw. Metal concentrations were right-skewed; thus, we log2-transformed them to minimize the impact of outliers and meet the normality assumption of linear regression. Harvard Pilgrim Health Care Institutional Review Board approved this cohort study. Participants provided written informed consent. We followed the STROBE reporting guideline and used R, version 4.1.0 (R Project for Statistical Computing) to analyze data from June to November 2023.

## Results

The sample included 368 mother-child pairs (mean [SD] maternal age, 33.1 [4.6] years; mean [SD] children’s age at blood draw, 3.4 [0.4] years; assigned sex at birth: female, 171; male, 197) with information on infant feeding practices and RBC metal concentration measurements (Table). Most children (92%) were fed human milk at some point over the first 6 months of life, 31% exclusively over that period, while 8% were never breastfed. At 6 months, 36% were fully weaned. Exclusive breastfeeding at 6 months vs never breastfed was associated with higher concentrations of magnesium (8.0%; 95% CI, 0.1%-16.4%), molybdenum (21.6%; 95% CI, 0.0%-47.8%), selenium (11.6%; 95% CI, 2.2%-21.9%), and arsenic (53.4%; 95% CI, 9.7%-114.4%) in blood during early childhood (Figure). Mixed feeding at 6 months vs never breastfed was associated with higher concentrations of copper (9.1%; 95% CI, 0.5%-18.5%), molybdenum (29.4%; 95% CI, 6.1%-57.8%), selenium (11.7%; 95% CI, 2.2%-22.2%), and barium (85.9% 95% CI, 2.9%-235.8%) (Figure).

**Table.** Characteristics of 368 Mother-Child Pairs With Infant Food Source and Early Childhood Red Blood Cell Metal Level Measurements Stratified by Food Source at 6 Months

Characteristic	No. (%)			
	Exclusive breastfeeding (n = 113)	Mixed feeding^a^ (n = 90)	Weaned from breastfeeding (n = 134)	Never breastfed (n = 31)
Maternal				
Age, mean (SD), y	33.6 (4.3)	33.4 (4.1)	32.5 (5.2)	32.8 (3.7)
Race/ethnicity^b^				
Asian	2 (1.8)	3 (3.3)	5 (3.7)	0
Black	6 (5.3)	9 (10.0)	24 (17.9)	5 (16.1)
Hispanic	3 (2.7)	5 (5.6)	9 (6.7)	1 (3.2)
White	99 (87.6)	72 (80.0)	90 (67.2)	25 (80.7)
>1 Race or ethnicity	3 (2.7)	1 (1.1)	6 (4.5)	0
Education level				
<College	18 (15.9)	16 (17.8)	44 (32.8)	16 (51.6)
College graduate	95 (84.1)	74 (82.2)	90 (67.2)	15 (48.4)
Annual household income				
≤$70 000	24 (21.2)	28 (31.1)	53 (39.6)	16 (51.6)
>$70 000	89 (78.8)	62 (68.9)	81 (60.5)	15 (48.4)
Parity				
Nulliparous	61 (54.0)	52 (57.8)	69 (51.5)	29 (93.6)
≥1	52 (46.0)	38 (42.2)	65 (48.5)	2 (6.5)
Child				
Assigned sex at birth				
Female	50 (44.3)	39 (43.3)	73 (54.9)	9 (29.0)
Male	63 (55.8)	51 (56.7)	61 (45.5)	22 (71.0)
Age at blood draw, y	3.3 (0.4)	3.4 (0.5)	3.4 (0.5)	3.4 (0.5)

^a^ Breast milk and formula.

^b^ Self-reported race and ethnicity were included as a proxy for shared differences in unmeasured variables such as racism and marginalization.

**Figure.**
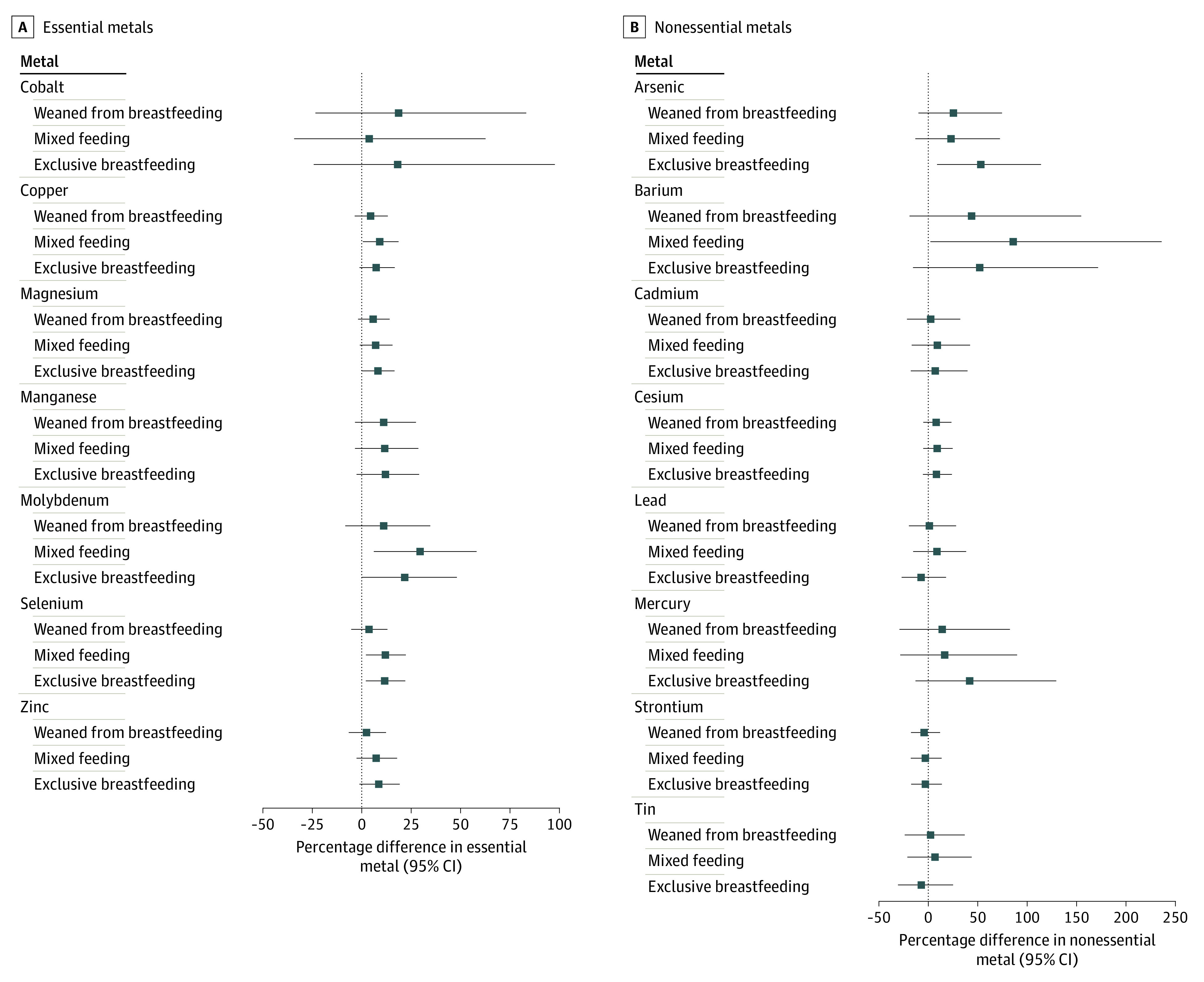
Association of Infant Feeding Practices With Essential and Nonessential Metal Concentrations in Children’s Blood Percentage difference for the association of maternal-reported infant milk source at 6 months (exclusive breastfeeding, mixed feeding [breastfeeding and formula], and fully weaned from breastfeeding) vs never breastfed, with concentrations of essential and nonessential metals in red blood cells during early childhood. Estimates are adjusted for maternal age at enrollment, race and ethnicity, education level, annual household income, parity, and children’s assigned sex at birth and age at blood metal level measurement.

## Discussion

Breastfeeding at 6 months was associated with higher concentrations of several essential metals, arsenic, and barium during early childhood. It is likely that blood arsenic levels reflect children’s fish consumption, as inorganic arsenic (toxic form) does not readily excrete into human milk. The association between mixed feeding and higher concentration of barium is consistent with a study that used barium-calcium ratio in dental tissues as a biomarker of infant dietary transitions.^4^

The associations may be related to differences in gut microbiota or the epigenome^5^ that affects metal metabolism and excretion^6^ and long-term storage. A study limitation was exclusion of food introduction timing and early childhood diet, which contribute to metal exposure but may be on the causal pathway. Our findings suggest that infant feeding practices may affect circulating blood metal levels several years later; therefore, studies examining infant and early childhood blood metal levels and health outcomes should account for infant feeding practices. Given implications of metal concentrations for neurodevelopment and growth, further research is needed on this exposure pathway.

## Notes

Supplement.
Data Sharing Statement
