# Supplementary material for: Infant Feeding Practices and Metal Concentrations in Children’s Blood
Source: JAMA Netw Open. 2023 Dec 18;6(12):e2348230. doi: 10.1001/jamanetworkopen.2023.48230 (PMC10728766; doi:10.1001/jamanetworkopen.2023.48230)
Supplement: Supplement. — Data Sharing Statement [file jamanetwopen-e2348230-s001.pdf]

Data Sharing Statement

Smith. Infant Feeding Practices and Metal Concentrations in Children's Blood. *JAMA Netw Open*. Published December 19, 2023. doi:10.1001/jamanetworkopen.2023.48230

Data
Data available: No

Additional Information
Explanation for why data not available: Because of the sensitive nature of the data collected for this study, requests to access the data set from qualified researchers trained in human subject confidentiality protocols may be sent to Project Viva at project_viva@hphc.org and to the corresponding author. Data can be accessed with the appropriate permission from the Project Viva study team following the study policies (https://www.hms.harvard.edu/viva/).
